# Sex differences in the shoulder joint position sense acuity: a cross-sectional study

**DOI:** 10.1186/s12891-015-0731-y

**Published:** 2015-09-30

**Authors:** Amir K. Vafadar, Julie N. Côté, Philippe S. Archambault

**Affiliations:** School of Physical and Occupational Therapy, McGill University and Interdisciplinary Research Center in Rehabilitation (CRIR), 3654 Promenade Sir William Osler, Montreal, QC H3G 1Y5 Canada; Department of Kinesiology and Physical Education, McGill University and Interdisciplinary Research Center in Rehabilitation (CRIR), 475 Pine Avenue West, Montreal, QC H2W 1S4 Canada

**Keywords:** Sex difference, Position sense, Shoulder joint, Motor control, Work-related musculoskeletal disorders

## Abstract

**Background:**

Work-related musculoskeletal disorders (WMSD) is the most expensive form of work disability. Female sex has been considered as an individual risk factor for the development of WMSD, specifically in the neck and shoulder region. One of the factors that might contribute to the higher injury rate in women is possible differences in neuromuscular control. Accordingly the purpose of this study was to estimate the effect of sex on shoulder joint position sense acuity (as a part of shoulder neuromuscular control) in healthy individuals.

**Methods:**

Twenty-eight healthy participants, 14 females and 14 males were recruited for this study. To test position sense acuity, subjects were asked to flex their dominant shoulder to one of the three pre-defined angle ranges (low, mid and high-ranges) with eyes closed, hold their arm in that position for three seconds, go back to the starting position and then immediately replicate the same joint flexion angle, while the difference between the reproduced and original angle was taken as the measure of position sense error. The errors were measured using Vicon motion capture system. Subjects reproduced nine positions in total (3 ranges × 3 trials each).

**Results:**

Calculation of absolute repositioning error (magnitude of error) showed no significant difference between men and women (p-value ≥ 0.05). However, the analysis of the direction of error (constant error) showed a significant difference between the sexes, as women tended to mostly overestimate the target, whereas men tended to both overestimate and underestimate the target (p-value ≤ 0.01, observed power = 0.79). The results also showed that men had a significantly more variable error, indicating more variability in their position sense, compared to women (p-value ≤ 0.05, observed power = 0.78).

**Discussion:**

Differences observed in the constant JPS error suggest that men and women might use different neuromuscular control strategies in the upper limb. In addition, higher JPS variability observed in men might be one of the factors that could contribute to their lower rate of musculoskeletal disorders, compared to women.

**Conclusions:**

The result of this study showed that shoulder position sense, as part of the neuromuscular control system, differs between men and women. This finding can help us better understand the reasons behind the higher rate of musculoskeletal disorders in women, especially in the working environments.

## Background

Work-related musculoskeletal disease (WMSD) accounts for over 33 % of all newly reported occupational illnesses in the general population in the United States, the Scandinavian countries and Japan [1] and is the most expensive form of work disability [2, 3]. The risk factors for the development of WMSD can generally be categorized as either physical or psychosocial [4]. Specifically for the WMSD in the neck and shoulder region, physical risk factors may include heavy physical work, awkward static and dynamic working posture and repetitive work; whereas psychosocial factors may include low level of work satisfaction and support, and high level of distress [5]. In addition, there are some individual risk factors that are shown to be associated with WMSD. Female sex is one of these factors that has become the focus of several epidemiological studies in the past few years [6, 7]. In the context of this paper, we will use the expression ‘sex’ vs. ‘gender’ in hypothesizing that our research object, position sense, is more so affected by biology than social constructs, although we recognize that both sex and gender may have an influence on our research and its context.

Previous studies have identified the presence of sex differences in the development of WMSD and have mostly reported a higher risk of injury in the same occupation in women compared to men. For example, experiments among army trainees, [8], semiconductor industry workers [9] and postal workers [10] have shown that women are at higher risk for musculoskeletal problems and occupational injuries. In particular, Larsson et al. [ 7 ] and Treaster & Burr [4] reviewed the existing literature and reported that women had a significantly higher prevalence than men for many types of shoulder and neck musculoskeletal disorders. When Treaster & Burr controlled for confounders such as age and work factors, the difference was still significant.

The higher prevalence of neck/shoulder WMSD in women can generally be attributed either to sex differences related to anthropometry and strength, or in aspects related to motor control [11] . The anthropometry and strength differences have been well described before. For example, it has been shown that men have a higher muscle strength and aerobic capacity [12] , exert more torque, work and power during functional work tasks [13] , and produce less effort and muscle contraction for the same task [14] , as compared to women. Specifically in the neck/shoulder region, it has been shown that women have weaker and smaller necks compared to men of similar height [15] , have a lower strength and 45 % less muscle cross-sectional area in the biceps brachii [16] and have a different shape and anatomy of glenoid fossa [17] . These differences may explain the higher rate of neck/shoulder WMSD in women. However, the recent findings of sex differences with respect to neuromuscular control suggest that factors other than anthropometry and strength may also contribute to the injury rate differences between sexes [18-20] .

Neuromuscular control refers to the control of the nervous system over muscle activation and the factors contributing to task performance [21]. This control is based on the integration of sensory information (i.e. proprioception, vestibular, visual) in the central nervous system (CNS), which can subsequently result in efferent responses to the muscular system [22]. The possibility that such a neural processing differ between men and women has been the focus of recent studies, with only a few of them focusing on the upper limb [18-20]. Specifically, possible sex differences in limb proprioception have received little attention. Proprioception is the afferent information that arises from mechanoreceptors in the muscles, joints and skin, and travels to the CNS to integrate with other sensory information [23]. In fact, proprioceptive information, along with other sensory information, provide a basis for neuromuscular responses. Pederson et al. [24] compared the kinesthesia (threshold to detect limb movement, a sub-modality of proprioception) of the shoulder joint between men and women and found that women’s ability to detect the initiation of movement was weaker than that of men. However, Bjorklund et al. [25] found no difference in shoulder position sense (a sub-modality of proprioception) between men and women before and after muscle fatigue. The same finding was reported by Emery and Cote [26], where no effect of sex on shoulder position sense was found after muscle fatigue. However the authors reported the existence of sex differences in the directionality characteristics of position sense, as women tended to systematically make repositioning errors anterior and inferior to the target, a pattern that was different from that of men.

Exploring possible differences in the proprioception and neuromuscular control between men and women can increase our knowledge about sex differences in the mechanisms of WMSD and eventually help to achieve better preventive strategies to decrease the higher rate of upper limb injuries in both sexes. Accordingly, the purpose of this study was to estimate the effect of sex on shoulder joint position sense acuity in healthy individuals. The position sense acuity was estimated based on the magnitude of shoulder repositioning error, the direction of error and the variability in the performance. We hypothesized that all these three factors would be different between men and women.

## Methods

### Design

A cross-sectional study design was used to compare the shoulder joint position sense acuity between men and women.

### Participants

Our sample size calculations estimated that a minimum sample size of 14 per group is required in order to observe at least 1 degree of difference in position sense between sexes, with the power of 0.8 and significant level of 0.05. Accordingly, 28 healthy participants from university students and employees, 14 females and 14 males, 25 right-handed and 3 left-handed (2 female, 1 male), mean age = 30, SD = 6.1, were recruited for this study. Participants did not have any problem in their shoulder girdle or neck, such as pain, limited range of motion (measured with goniometer), or any recent or previous history of injury (including previous surgeries) in the upper extremity in the past year. Subjects were not routinely pursuing any activity that required continuous function of the shoulder joint (such as playing sports or playing musical instruments that required shoulder joint movements). In addition, subjects’ occupation did not involve continuous function of the shoulder (17 graduate students, 6 undergraduate students, 5 office jobs). The protocol of the study was approved by the ethics board of the *Center for Interdisciplinary Research in Rehabilitation (CRIR)* in Montreal, Canada. All subjects provided their informed consent using forms approved by the ethics board, prior to the experiment.

### Procedure

For the assessment of position sense, subjects were asked to actively reproduce different shoulder flexion angles with their dominant arm, within three different target ranges (55 ± 10° (low-range), 90 ± 10° (mid-range) and 125 ± 10° (high-range)), three times each. Therefore, subjects were not reproducing specific, pre-determined shoulder flexion angles (e.g. *exactly* 55°, 90° or 125°). Instead, they were asked to reproduce their previous shoulder angle, which could fall anywhere within the three ±10° target ranges. With this procedure, we prevented the subjects from learning three specific angles, as we wanted them to reproduce their previously reached shoulder angle instead of fixed shoulder angles. Position sense was tested during shoulder flexion because this is the most common movement of the arm during daily activities, especially during reaching movements.

Participants initially stood still with their arms resting alongside of their body and with their eyes blindfolded. At a voice command, they started to flex their dominant shoulder at a comfortable speed while their elbow remained fully extended and with their forearm and wrist in neutral position (thumb facing upward). When participants reached one of the predefined target ranges (selected in random order), the experimenter asked them to stop, then to maintain their shoulder flexion angle for three seconds. They were then asked to bring back their arm to the starting position and then to immediately reproduce the previously reached shoulder angle. Subjects were asked to let the experimenter know when they felt that they had reached the remembered angle. The instructions to the subject were to “flex the shoulder until you are told to stop” for the first movement and “replicate the same shoulder angle” for the replication tasks. Subjects reproduced nine angles in total (3 ranges × 3 trials each). Each trial lasted about 20 seconds. Subjects practiced a trial once before the beginning of the experiment. Subjects did not have any break during the 9 repositioning tasks.

### Outcome measure

The main outcome of this study was shoulder position sense, which was measured using a Vicon motion capture system (VICON©, Oxford Metrics ltd., Oxford, UK). A series of passive, reflective markers were fixed on principal anatomical landmarks of the upper limb and trunk according to the Vicon Plug-In-Gait upper extremity model. These landmarks were: spinous processes of C7 and T10, manubrium and xiphoid process of sternum, left and right acromion and right lateral epicondyle of humerus. Vicon has been shown to be valid (*r = 0.94*) and have a very high repeatability (0.78°) in the measurement of shoulder joint flexion angle [27]. Therefore it was sensitive to detect at least 1 degree of difference in joint position sense that was used in our sample size calculations.

### Analysis

For calculation of shoulder joint angle, we first defined the arm as a vector based on the position of the markers on the shoulder and elbow. In order to calculate a pure flexion angle in one plane (sagittal plane) and eliminate the abduction component of the movement, we defined the projection of the arm vector onto the true sagittal and frontal planes. We then defined the shoulder flexion angle by calculating the angle between the arm vector in the sagittal plane and the true frontal plane in a two-dimensional space.

Subjects’ repositioning accuracy was evaluated using three types of error: 1) *absolute error*, i.e. the measure of the magnitude of the error, discounting direction; 2) *constant error*, i.e. the measure of the deviation from the target and 3) *variable error*, i.e. the measure of the consistency in performance. Although most of the previous studies have measured joint position sense as the absolute repositioning error [25, 28]., Janwantanakul et al. [29] suggest that the magnitude of error (absolute error) alone may not provide an accurate representation of the subjects’ position sense ability and that consistency in performance (variable error) should also be considered when evaluating joint position sense. As an example given by the authors, if a subject produces an absolute error of 10° for each of the three trials, he/she would have an average absolute error of 10° and a variable error of 0°; whereas if she/he produces absolute errors of 5, 10 and 15°, there would still be an average absolute error of 10°, but a variable error of 5°. In fact, unlike the absolute error, variable and constant errors might reflect how accurately the target is represented in the nervous system and provide a different information on the integrity of the sensorimotor system [30]. In this study, constant error value was calculated by taking the difference between the reproduced and the reference angle:

1 $$ {E}_{constant}=\left({X}_i\mathit{\hbox{-}}T\right)/N $$

where xi was the produced angle, T the reference angle and N the number of trials, calculated separately for each target range.

For absolute error, the absolute value of the difference between reproduced and actual angle was used:

2 $$ {E}_{absolute}=\left|{X}_i\mathit{\hbox{-}}T\right|/N $$

Constant and absolute errors made by female and male participants were then compared using a two-way ANOVA (2 sexes × 3 target ranges). If significant differences were found, a post-hoc analysis (Tukey HSD) was performed.

The variable error was calculated as the overall standard deviation (SD) of constant error from 9 trials, irrespective of the target range:

3 $$ {E}_{variable}=\sqrt{{\displaystyle \sum {\left({X}_i\mathit{\hbox{-}}M\right)}^2}} $$

where M was the average constant error over all trials.

The variable error for the male and female participants was then compared using an independent t-test. Processing of Vicon data was performed using MATLAB 8.3, The Mathwoks, Inc., Natick, Massachusetts, USA. Statistical analysis was performed using SPSS, version 22.

## Results

**Fig. 1:**
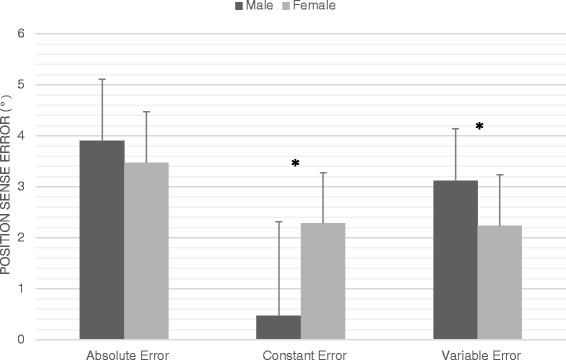
Overall position sense error in the shoulder joint. Comparison of absolute, constant and variable errors between men and women considering all three target ranges * *p-value ≤ 0.05*

Repositioning errors made by each sex in each shoulder range are shown in Table 1. Subjects’ average movement speed was 0.21 ± 0.12 m/s. Considering the absolute repositioning error, the results showed a significant difference between the ranges (p < 0.001), with the post-hoc analysis showing a significantly larger error in the early compared to the mid (p < 0.001) and high-ranges (p < 0.001), but no difference between mid and high-ranges (p = 0.97), for both sexes. However, no significant interactions between the sex and shoulder ranges were observed. The results showed no absolute position sense error differences between men and women (3.9° vs. 3.4° respectively, p = 0.38). The analysis of the direction of error (constant error) showed a significant difference between all three shoulder ranges for both men and women (F(2, 78) = 17.07, p < 0.001, observed power = 0.99) (Table 1), but no significant interaction between sex and shoulder ranges. The results also indicated a significant effect of sex on the direction of error (constant error) as women tended to mostly overestimate the target (81 % of the time), whereas men tended to both overestimate (55 % of the time) and underestimate (45 % of the time) the target (2.28° vs. 0.47° respectively, F(1, 78) = 8.0, p = 0.006, observed power = 0.79). Finally, the results showed that men had a significantly larger variable error compared to women (3.12° vs. 2.24° respectively, p = 0.02, observed power = 0.78) (Fig. 1).

**Table 1:** Absolute, constant and variable errors made by men and women in each shoulder range. Only overall value is presented for variable error

Absolute error		
Shoulder ranges	Female	Male
Low-range	5.01 ± 2.65°	5.65 ± 2.59°
(55 ± 10°)		
Mid-range	2.8 ± 1.28°	3.4 ± 1.58°
(90 ± 10°)		
High-range	3.11 ± 1.22°	2.93 ± 1.4°
(125 ± 10°)		
Constant error		
Shoulder ranges	Female	Male
Low-range	4.23 ± 2.89°	3.34 ± 4.02°
(55 ± 10°)		
Mid-range	2.63 ± 1.7°	−0.18 ± 2.93°
(90 ± 10°)		
High-range	0.03 ± 2.62°	−1.58 ± 2.07°
(125 ± 10°)		
Variable error		
Shoulder ranges	Female	Male
Overall	2.24 ± 0.77°	3.12 ± 1.02°

Position sense error in each shoulder range

## Discussion

The results of this study was consistent with the findings of the previous studies [25, 28] showing no significant difference between men and women when position sense was measured as absolute repositioning error. However, our findings showed a significant difference in constant (direction-specific) position sense error between men and women (i.e. fewer men overshot the target) and a significantly higher variability in the performance of men compared to women. Although a few factors could potentially influence joint position sense (e.g. active or passive movements, muscle fatigue [31] and gravity [32]), we made sure in this study that all of these factors were equal for both men and women subjects so that the significant difference observed was in fact due to the effect of sex.

The findings of this experiment showed that both sexes made lower position sense error in the mid and high-ranges, and higher error in the low-range. We have previously discussed in details the possible explanations for the better shoulder position sense in the mid and high-ranges [33]. It seems that the lower JPS error observed in our study was primarily due to the higher activity of musculotendinous mechanoreceptors (GTO and muscle spindle) in these ranges, where a stronger muscle contraction was needed to hold the arm against gravity [32].

The results of this study showed that women tended to mostly overestimate the target, whereas men tended to both overestimate and underestimate the target, resulting in a lower constant error and a higher variability in men compared to women. Such a difference in the direction of error has also been reported by Emery and Cote [26], where the authors found that during a reaching task after muscle fatigue, women tended to systematically make errors that were more anterior and inferior of the target, different from errors made by men. It is not very clear why the direction of error was different in our experiment, but one possible reason could be the different strategies that men and women employ during movement tasks. It has been suggested that women may use different strategies and work methods than men when performing physically demanding work [34]. Rohr [19] reported that during a pointing task performed on a computer, women used strategies that relied more on precision, while men used strategies that relied on movement initialization and response speed. It seems that in our study, women tended to use a constant motor control strategy which made them constantly overestimate the target, whereas men probably used more than one motor control strategy during shoulder repositioning task, which resulted in their more random performance.

Employing different repositioning strategies by men caused them to also have a significantly higher variable error compared to women. Variability is a common feature of human movement that may play a role in the central organization of voluntary movement [35]. Previous studies have tried to distinguish between variability that has no effect on the quality of performance (“good” variability) and variability that has negative effect on the performance (“bad” variability) [36]. In fact, the presence of a level of variability in body movements is essential to make the movements more flexible and stable (good variability) [37]. However, when variability increases beyond its optimal value, it makes the neuromuscular system noisier and less adaptable [38], and when it decreases below its optimal value, the beneficial effects of redundancy in the motor system is decreased [39]. Therefore both conditions, variability beyond and below the optimal value, can increase the chance of injury. Possible effects of sex on motor variability have also been the focus of a few studies. Svendsen and Madeline [40] studied sex differences with respect to variability and reported that during an elbow flexion endurance task, women tended to be less variable in force production compared to men. Fedorowich et al. [41] found that women who showed a higher initial variability in the electromyography (EMG) signal of upper trapezius and supraspinatus muscles had a higher endurance during upper limb fatiguing exercises, which could potentially reduce the risk of injury. The result of our study showed that men had a higher shoulder repositioning variability compared to women. These results, along with the previous findings, can help us achieve a better understanding of the causes of higher WMSD rate in women and to employ preventive strategies to decrease the risk of injury. Although the observed difference in the constant and variable errors between sexes was minimal (1 to 2 degrees), the presence of such a difference by itself seems to be more important than the size of the difference. The fact that women overshot the target in 81 % of the time compared to 55 % of the time in men could be suggestive of an important difference in the neuromuscular control system between sexes. This is especially important in WMSD, where injuries are mostly caused by minor traumas over a long period of time. Employing a constant movement strategy with less variability in performance by women might put some body structures under a minimal but long-term stress, which might eventually lead to a significant injury.

One of the preventive strategies to decrease the chance of injury that could be the interest of clinicians is to manipulate the motor variability by training or educating individuals in a way that they perform their work with a proper motor variability [42]. Although no studies have yet investigated if motor variability can be changed by training in a specific manner, a few experiments have demonstrated that it is possible to selectively activate different neuromuscular compartment within the muscles of the neck and upper arm, specifically the trapezius muscle [43, 44]. Future investigation on the possibility of manipulating motor variability could help clinicians in providing training aimed at optimizing motor variability through specific exercises.

### Limitation of study

This study was limited to the movements of the shoulder in the direction of flexion. It also focused solely on shoulder position sense, a sub-modality of proprioception, and representing a single aspect of the motor control system. Further studies are needed to achieve a better understanding of s/g differences with respect to motor control.

## Conclusion

The result of this study showed no s/g differences in shoulder position sense when it was estimated as absolute error. However, the results indicated that the direction of error was significantly different between men and women, and men’s performance tended to be significantly more variable than that of women. This finding can help us better understand the reasons behind the higher rate of WMSD in women.

## Abbreviations

WMSD: Work-related musculoskeletal disease
CNS: Central nervous system
SD: Standard deviation
CI: Confidence interval
